# Relationship between Socio-Demographic Factors and Posttraumatic Stress Disorder: A Cross Sectional Study among Civilian Participants’ Hostilities in Ukraine

**DOI:** 10.3390/ijerph19052720

**Published:** 2022-02-26

**Authors:** Stanisław Fel, Krzysztof Jurek, Katarzyna Lenart-Kłoś

**Affiliations:** The Institute of Sociological Sciences, The John Paul II Catholic University of Lublin, 20-950 Lublin, Poland; stanfel@kul.lublin.pl (S.F.); katarzyna.lenart-klos@kul.pl (K.L.-K.)

**Keywords:** posttraumatic stress disorder, social and demographic factors, hostilities in Ukraine

## Abstract

Background: Even though there is an extensive body of literature on posttraumatic stress disorder (PTSD) in individuals who have experienced armed conflict, there are still many grey areas, especially in relation to civilian participants in hostilities. This article evaluates how socio-demographic factors and the interactions between them have influenced PTSD among civilians involved in the recent war in Ukraine. Methods: This cross-sectional study included a convenience sample of 314 adults, 74 women, and 235 men. The mean age was 34.08 years. We used the Posttraumatic Stress Disorder (PTSD) Checklist–Civilian Version (PCL-C). Results: Our findings show that predictors of posttraumatic stress are loss of a loved one, place of residence, gender, continuation of education, and health insurance. We demonstrated that PTSD produced by the loss of a loved one as a result of war is determined by participation in the education system, whatever the level of education. The literature emphasises the importance of social support, e.g., from the family. We demonstrated that having children is associated with a risk of more severe PTSD, causing serious mental strain among participants of hostilities. We discovered that material security lowers PTSD, but only among people who have no children. Conclusions: PTSD is the result of not only the violence and damage caused by war but also of other stressful circumstances associated with the social and financial conditions of life. Further research needs to focus on identifying modifiable risk factors and protective factors that could be embraced by intervention strategies. Our findings can inform the goals behind therapeutic support for civilian participants of hostilities, and implications for social work. Social work professionals are encouraged to engage in direct questioning and to maintain a supportive and safe environment for participants in hostilities, e.g., in the area of education. Trauma-affected people need to be given opportunities to build up their strengths and increase their psychological resources towards well-being. Social security (health insurance, savings, material security) should be taken into account when working with people affected by PTSD.

## 1. Introduction

It is estimated that, from the end of the Cold War, nearly half of all countries in the world have been affected by armed conflict. Hoppen and Morina [1] argue that between 1989 and 2015 nearly a billion people lived in regions directly involved in war. Based on their research, they estimated that about 354 million adults, who have experienced war, suffer from posttraumatic stress disorder (PTSD) or major depression (MD), and approximately 117 million of them suffer from both these conditions.

### 1.1. Hostilities and PTSD

It is important to note that the main arena of war operations (e.g., Russia’s war operations in Ukraine) is not in the physical space, but in the psychological one. Rather than physically annihilating the enemy and taking control of its territory, war operations are designed to break the enemy’s fighting spirit and reduce its resistance. In contemporary war operations, the distinction between soldiers and civilians is blurred [2]. Previous, traditional, approaches to warfare, focusing mainly on military action, are now being replaced by different types of conflict. New kinds of war are emerging. These include hybrid warfare, asymmetric warfare, new generation warfare, non-linear warfare, controlled chaos, and low-intensity conflict. Hostilities are one of the biggest stressors and often lead to serious mental health problems, including PTSD. PTSD is described as a delayed and/or prolonged reaction to highly stressful events which are associated with loss of life, serious bodily harm, and/or danger to the physical integrity of oneself, accompanied by intense anxiety, feeling of helplessness, and/or danger. PTSD is characterised by such symptoms as recurring images, dreams, thoughts related to the experienced event (intrusion), attempts to avoid thinking about the situation and its experiences (avoidance), manifesting aroused vigilance, fear, difficulty concentrating and/or overstimulation [3,4]. The changes may affect the person’s psychological, behavioural, and physical health [5]. In addition, PTSD often co-occurs with depression, anxiety, substance abuse, and suicidal behaviours [6]. The direct effects of PTSD concern intimate relationships, such as marriage, social interactions, decreased productivity, and decreased fitness. Generally, the longer PTSD remains untreated, the greater the probability that this stress-related disorder will lead to a health complication [7].

### 1.2. Rates of PTSD

In a study by Hoge et al. [8] PTSD was diagnosed in 18% of Iranians and 11.5% of Afghanistan war veterans. Hall et al. [9] reported PTSD in 26% of the Gaza population. Ahern et al. [10] reported 7.4% of emergency department patients, 2 years after the end of the war in Kosovo, had experienced traumatic events, and 89.5% had posttraumatic stress symptoms. Groth et al. [11] found PTSD in 56% of soldiers who had taken part in peacekeeping missions in Iraq or Afghanistan. In a study conducted among adult war refugees and migrants, the prevalence of PTSD was between 2.2% and 88.3% [12,13]. Among the refugees from Cambodia displaced to the USA and participating in the research, 61% of them were found to have PTSD [14]. In the National Vietnam Veterans Readjustment Study (NVVRS) researchers found that 33% of male veterans with PTSD reported intimate partner aggression in the previous year, compared to 13.5% of those without PTSD [15]. According to the study conducted on a representative sample of the adult Rohingya refugee population in camps in Bangladesh, 61.2% of participants admitted to suffering from posttraumatic stress symptoms typical of PTSD [16].

### 1.3. Risk Factors for PTSD

The above statistics are approximate since many people in need of treatment do not seek care. For a military population, one of the most important risk factors for the onset of PTSD is exposure to combat [17], being wounded or injured [18], witnessing death [19], or being tortured or being taken captive [20]. PTSD is associated with a personal life-threatening assessment during trauma, lower rank in the military hierarchy, low morale, poor social support, and non-receipt of a homecoming brief [21]. Other factors impacting on the increased risk of PTSD in the veteran population are younger age at the time of the trauma, racial minority status, lower socio-economic status, lower education, higher number of deployments, longer deployments, prior psychological problems, lack of social support, lower military rank, and ethnicity [22]. It turns out that anger and hostility are both higher in people with PTSD than in people without PTSD, and they are significantly higher in those suffering from PTSD associated with military experience [23]. The health consequences of hostilities extend beyond death, disease, and trauma-related psychiatric illness, including the pervasive effects of the destruction of economic and social institutions and the structure of society as a whole. Capacity-building activities should prioritise health care and social work staff who are most needed by traumatised individuals.

The results presented in the article represent a voice in the ongoing discussion in the literature on the factors producing PTSD among civilian populations caught up in the hostilities in Ukraine. On the one hand, this study expands the existing knowledge on the factors contributing to PTSD, while on the other hand, it provides new insights to advance our understanding of the relationship between socio-demographic factors and severity of PTSD. These findings can be used by social workers who provide support to civilians experiencing trauma as a result of war. This manuscript aims to contribute to the current literature by (1) examining the impact of socio-demographic factors on PTSD among civilian participants in the hostilities in Ukraine, (2) identifying the factors behind lower PTSD, and (3) analysing interactions between selected predictors of PTSD.

## 2. Materials and Methods

The study was conducted in 2019 among Ukrainian civilians living in towns located in the Donbass, a region in eastern Ukraine, where an armed conflict has been going on since 2014 between pro-Russian separatists and the Russian Federation supporting them, and the army representing the legal authorities of Ukraine. Displaced people from Donbas, who were temporarily living in the central or western parts of Ukraine, also participated in the research.

### 2.1. Participants and Procedure

A convenience sampling method was used. The research was carried out in hospitals, readaptation centres, displaced persons’ places of stay. Answers were given in the presence of trained interviewers of Ukrainian nationality. The supervisors of the survey were employees of the Institute of Psychology and the Institute of Sociological Sciences at the John Paul II Catholic University of Lublin.

Our quantitative cross-sectional study included 314 adults, 74 women and 235 men (5 respondents did not indicate their gender), aged between 18 and 74. The mean age was 34.08 (*SD* = 9.83).

Participants were fully briefed on the aim of the study, and their queries were answered by researchers. The study was anonymous. The procedure was approved by the Research Ethics Committee of the Institute of Sociological Sciences of the John Paul II Catholic University of Lublin (protocol code: KEB-IS-3/2019).

### 2.2. Measure

The PTSD Checklist [24] is a 17-item scale originally based on the DSM-III-R posttraumatic stress disorder criteria and revised in 1994 to correspond to the DSM-IV criteria. Respondents indicate how much they were bothered by each PTSD symptom in the past month (5-point scale: 0—means not at all; 4—means often). Cronbach’s alpha for the whole scale was 0.97. A PCL-C total score was calculated by summing each of the 17 items, with higher scores indicating higher levels of PTSD symptom severity (range 0–68). A diagnosis of PTSD was determined when an individual met DSM-IV symptom criterion defined by the presence of at least 1 B item (questions 1–5), 3 C items (questions 6–12), and at least 2 D items (questions 13–17). Symptoms rated as “Moderately” or above (responses 2 through 4 on individual items) were counted as present. We used the civil version of the tool (PCL-C) to allow respondents to report PTSD symptoms for any traumatic events, not only symptoms produced by war experiences. In the evaluation of the overall mental health of participants in war, it is particularly important for the assessment of PTSD symptoms to include both military and non-military sources of trauma.

### 2.3. Statistical Methods

Data were analyzed using SPSS.25. Univariate and multivariate analyses were used to assess determinants of PTSD. The comparisons between groups were done using parametric tests: independent-sample t-tests were conducted to compare PTSD scores for independent variables with two levels (e.g., gender), and one-way between-group analyses of variance (ANOVA) were conducted to compare the effects of the independent variables with more than two levels in PTSD score. Two-way ANOVA was used to estimate how the mean of PTSD score changes according to the levels of two categorical variables. We assessed skewness and kurtosis. If the data were greater than +1 or less than −1, and for kurtosis, the data were greater than +1, the data distribution generally deviated from a normal distribution. Deviations and normality were checked with the Shapiro–Wilk test. Homogeneity of variance was assessed using Levene’s test of equality of variance. The effect sizes were computed. The Cohen [25] guidelines were followed to interpret the values. Multiple linear regression analysis (the stepwise method) was used to identify independent variables (socio-demographic characteristics) which predicted PTSD outcomes. The assumptions of linearity and homogeneity of variance were checked using scatter plots and no heteroscedasticity/no clear pattern was found in the plots. Skewness was within ±1. Multicollinearity was checked and the minimum and maximum variable inflation factor (VIF) were 1.045 and 1.124, respectively, indicating that there was no risk of multicollinearity. A general F-test and an adjusted R-square were considered. Standardized Beta coefficients (β) were calculated to assess the level of association and statistical significance in the multiple regression analysis.

The obtained results of the analysis were assumed to be statistically significant at *p* < 0.05.

## 3. Results

The average PTSD score was 20.7 (*M* = 21.0; *SD* = 13.87). PTSD was diagnosed in 37.3% of Ukrainians. Statistically significant differences were found in PTSD scores based on age, gender, having children, continuing education, place of residence, financial situation, lost loved one, and health insurance (Table 1). No significant differences were identified in PTSD scores (*p* > 0.05) according to civil status, level of education, distance from hostilities, savings, and financial security.

**Figure 1:**
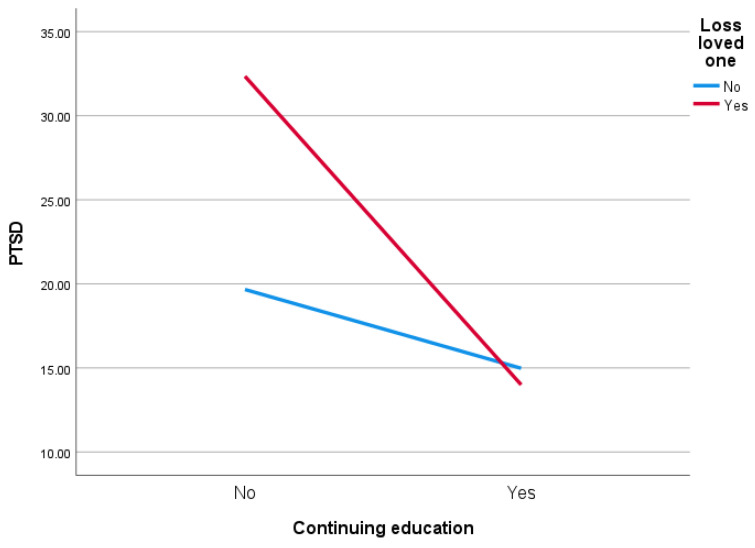
Effect of loss of loved one and continuing education on PTSD score.

A two-way ANOVA showed the main effects and the interaction effect in the two models. The first model accounted for the loss of a loved one (Yes vs. No) and continuing education (Yes vs. No). The residuals had a normal distribution. Levene’s test of equality of error variance also showed that the assumption of homogeneity of variance was not violated (*F* = 1.337; *p* = 0.262). The main effect was obtained for lost loved one (*F* = 5.622; *p* = 0.018; eta squared = 0.02, small effect size). The main effect of continuing education was statistically significant (*F* = 21.762; *p* < 0.001; eta squared = 0.07, medium effect size). The interaction effect was also statistically significant (*F* = 7.670; *p* = 0.006; eta squared = 0.03, small effect size). Participants in hostilities who had lost a loved one and continued their education had lower PTSD than people who did not continue their education (Figure 1).

**Figure 2:**
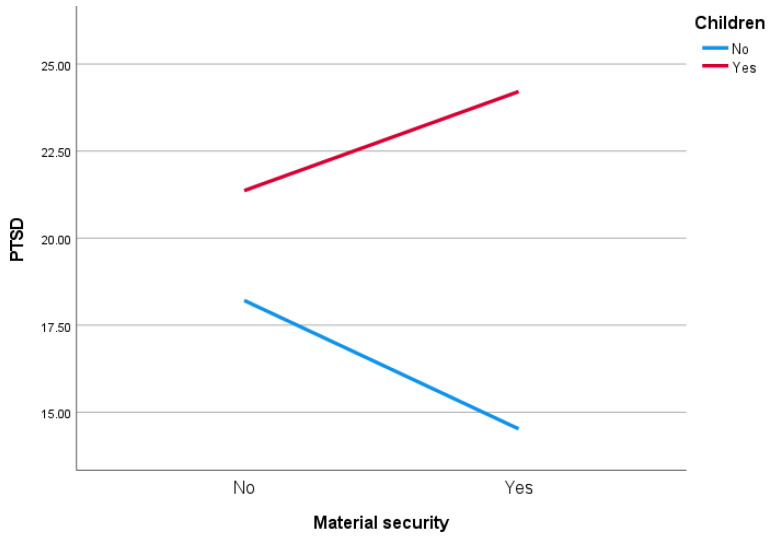
Effect of having children and material security on PTSD score.

In the second model, the variables were material security (Yes vs. No) and having children (Yes vs. No). The residuals had a normal distribution. The assumption of homogeneity of variance was confirmed (*F* = 1.353; *p* = 0.257). The main effect was not obtained for material security (*F* = 0.067; *p* = 0.796). The main effect of having children was statistically significant (*F* = 15,439; *p* < 0.001; eta squared = 0.05, medium effect size). The interaction effect was also statistically significant (*F* = 3.994; *p* = 0.047; eta squared = 0.01, small effect size). Participants in hostilities who had material security and children were characterised by higher PTSD than people who had no children (Figure 2).

### Predictors of PTSD

A multiple linear regression was carried out to examine potential PTSD predictors using the stepwise method (introducing 8 variables into the model: age, gender, having children, continuing education, place of residence, financial situation, lost loved one, and health insurance). The final model (*F* (5, 267) = 18.529, *p* < *0*.001) predicted 26% of the sample outcome variance with a coefficient of determination (Adj. *R*^2^ = 0.24). The model fulfils homoscedasticity criteria, and the residues are normally distributed (Table 2).

The resulting model contains five significant PTSD predictors: lost loved one (*β* = 0.208, *t* = 3.867, *p* <.001), place of residence (*β* = −0.280, *t* = −5.126, *p* <.001), gender (*β* = 0.270, *t* = 4.821, *p* <0.001), continuing education (*β* = −0.185, *t* = −3.389, *p* = 0.001), and health insurance (*β* = −0.172, *t* = −3.118, *p* = 0.002). The model predicted lower PTSD for participants in hostilities who lived in cities, continued their education and having health insurance. Higher PTSD correlated with lost loved one, and being a female (Table 3).

## 4. Discussion

PTSD is considered to be among the most prevalent mental disorders in populations affected by war, and much more prevalent compared to the communities that have not been recently involved in any conflict [26]. The loss of certain resources by populations participating in armed conflict, including soldiers, civilians, and displaced people, is associated with a higher risk of stress and/or depression [27,28,29].

### 4.1. Loss of a Loved One as a Predictor of PTSD

In this study, we made an attempt at identifying the variables that would determine PTSD levels in populations affected by the recent war in Ukraine. The loss of a loved one proved to be a predictor of higher PTSD. An unexpected death of a loved one is the most frequently reported traumatic experience in epidemiological studies worldwide [30]. This is true for various age groups. The results of research among teenagers who lived in the former Yugoslavia during the civil war between 1991 and 1995 show that their most traumatic experience was the loss of a family member as a result of military operations [31]. The loss of a loved one can produce what is known as prolonged grief disorder (PGD). Heeke, Kampisiou, Niemeyer, and Knaevelsrud [32] demonstrated a correlation between PGD and being a female, having a low level education, ruminating, having lost reasonably close relatives, and avoiding attachment, as well as an increased risk of co-morbidities such as depression and PTSD. Research conducted among U.S. military veterans has shown that the loss of a loved one aggravates the condition of people suffering from PTSD. It can increase social isolation and loneliness, or exacerbate PTSD symptoms [33]. However, not only the loss of a loved one can contribute to increasing PTSD. War experiences or the need to emigrate as a result of war, and the associated constant fear for the lives or safety of your loved ones, can also constitute predictors of PTSD [34].

### 4.2. Place of Residence as a Predictor of PTSD

Another predictor of PTSD is the place of residence. Living in a city proved to be correlated with lower PTSD. Research on combat veterans in the United States showed higher PTSD levels in veterans living in rural areas [35]. Similar results were obtained for Latino veterans living in the countryside [36]. Living in a city can be associated with access to various forms of support, e.g., from public institutions and NGOs. Therefore, lower PTSD among people living in cities can be connected to more support opportunities, e.g., in terms of employment, access to healthcare, etc. Generally, support helps mitigate psychological stress [37,38]. Research shows a positive impact of social support on reducing the risk of PTSD among prisoners of war [39], and among Albanian civilians in Kosovo following the civil war in the Balkans [1].

### 4.3. Continuing Education as a Predictor of PTSD

Continuing education proved to be another predictor of PTSD. A meta-analysis of 32 studies examining risk factors for PTSD showed a correlation between PTSD and lower levels of education [40]. In regions affected by armed conflict, emergency (e.g., natural disaster), or crisis, both children and adults tend to perceive schools as places of refuge and education, and a path to a better future. International organisations, such as Save the Children, have considered the involvement of children in education as a priority for children, and have called upon both national governments and humanitarian organisations to provide children with access to education. Schools provide the necessary mental health support [41]. The United Nations Girls’ Education Initiative in the East Asia Pacific Region, which seeks to provide both boys and girls with primary and secondary education, has argued there is a need to provide additional support to people who have experienced trauma. What proved relevant in our study was not so much having education as continuing education. This suggests the need to take measures to support continuing education also among adult participants in war. Entering or continuing education enhances resilience and resistance to external factors, supports social and emotional development, and gives hope for a better future. Our study shows an interaction between the loss of a loved one and continuing education. In the group of people continuing education, PTSD levels were similar among the respondents who had lost a loved one and those who had not had such war-related experience. For people who had not continued their education, PTSD levels were much higher among people who had lost a loved one compared to those who had not lost a loved one as a result of war. Through education, communities are able to undo the damage, recover from some traumas, and, in the long term, build communities based on peace. Being able to continue your education can also help you overcome trauma by, e.g., boosting your self-confidence and teaching you how to control your emotions and build relationships based on trust [42,43]. The opportunity to continue learning can be seen as a subjective resource that feeds into the resource increment spiral, according to S. Hobfoll’s COR concept (COR). Traumatic functioning is associated with high levels of resource distribution, mainly multidimensional losses, and less frequently with perceptions of resource gains. Categories of resource distribution are in turn indicative of posttraumatic change. Experienced losses increase the risk of negative outcomes (e.g., depression), while perceived gains increase the likelihood of experiencing development/posttraumatic growth in different life domains [44]. A learning opportunity can therefore be understood as a resource that can help people cope with a lack of resources in another area. Continuing to learn, learning can contribute to strengthening one’s self-esteem, the feeling that life has meaning, the ability to set goals, etc. Moreover, it is a source of instrumental, material, or value support. Among traumatized individuals, a sense of inferiority hinders their capability to receive social and emotional support and thus leads to feelings of loneliness [45]. Our study shows that with the loss of a loved one, the ability to continue learning becomes even more important for those experiencing armed conflict, and this problem affects not only children and adolescents but also adults.

### 4.4. Gender as a Predictor of PTSD

An important predictor of PTSD is being a female. Higher PTSD in women was reported in studies on adults exposed to traumatic events in Gaza [46], on Congo refugees [47], among Rwanda and Somali refugees living in Uganda [48], among Iranian Yazidis displaced to Turkey [49], among navy and marine soldiers after their return from operations in Iraq, Afghanistan or Kuwait in 2008 and 2009 [50], and soldiers involved in operations in Afghanistan and Iraq [51]. Studies on civilian populations have also consistently demonstrated that, compared to men, women show much higher levels of depression, anxiety, and PTSD [52]. A study conducted in 2016 on internally displaced persons (IDPs) in Ukraine also showed that women were more likely to suffer from PTSD, depression, and anxiety [53]. However, the results are not consistent. Haskell et al. [54] demonstrated that military service in Iraq was associated with a lower risk of PTSD in women compared to men. Nevertheless, one of its hypotheses suggests that this risk had more to do with the intensity and frequency of combat experience than with gender. The National Vietnam Veterans Readjustment Study also shows a higher prevalence of PTSD among men—Vietnam veterans [55]. The other study of male and female veterans found that PTSD symptoms predicted future drug use problems among male veterans, while drug use problems predicted future increases in PTSD symptoms among women [56]. In our study, higher levels of PTSD correlated with being female.

### 4.5. Lack of Health Insurance as a Predictor of PTSD

Yet another significant predictor of higher PTSD is the lack of health insurance. Lack of insurance proved to negatively affect the sense of security in relation to both oneself and one’s family members. A study carried out among US veterans has shown that difficulties in access to the healthcare system are a factor contributing to the high incidence of suicide among veterans suffering from depression and PTSD [57]. Prevalence rates for mental illness and trauma are disproportionately high among American veterans, especially those of the recent wars in Iraq and Afghanistan. Low-income individuals cannot afford additional health insurance which would provide comprehensive mental health coverage to meet their own needs and the needs of their family members who might experience depression, anxiety, and/or PTSD. Negative consequences of this include substance abuse, poor coping, difficulty in managing negative emotions, etc. [58]. Veterans need to rely on public programmes. Research among Palestinian teenagers has shown that low socio-economic status creates a risk of increased PTSD because economic hardship makes it difficult to access healthcare and get insurance due to limited financial resources [59].

### 4.6. Implications

The above findings suggest that PTSD prevention and treatment should be made a public health priority [60]. What we need is the development of military social work so that specialised support personnel provides professional assistance to veterans and civilians who suffer in the aftermath of war, especially in terms of health. Appropriate training of social workers in military social work could contribute to the development of services provided by institutions that offer support to individuals experiencing post-war trauma [61]. It has been emphasised that military social workers providing front-line support to soldiers, enhance their well-being and help them return to normal life after the war [62,63]. Actions to support mental health, as taken by military social workers in relation to individuals experiencing war trauma, should also cover civilian populations, who are also caught up in the hostilities, often against their will. The need to do social work with people who have experienced armed conflict was addressed in a few publications [64,65,66]. Steps taken as part of social work, in its broad sense, on territories affected by wars, or with populations displaced as a result of war, should engage individuals and communities as a whole to empower them and to support their rights [66]. An important area of social work with war-affected individuals is taking a broader look at the family as a provider of support for coping with traumatic experiences, and making interventions or developing programmes to address the needs of individuals and families as a whole [67,68] so that PTSD does not affect their loved ones [69].

PTSD is the result of not only the violence and damage caused by war but also of other stressful circumstances associated with the social and financial conditions of life. Further research to identify modifiable risk factors and protective factors that could be included in intervention strategies for PTSD prevention and treatment could rely on ecological models. Such models offer multi-dimensional approaches to recovery after trauma and suggest that the effectiveness of trauma interventions depends on the degree to which these strengthen the relationship between the individual and the community. Encouraging prosocial behaviour within local communities can produce favourable changes [70]. PTSD is, therefore, the product of social, economic, and cultural conditions, which can be regulated through appropriate public health policies [59]. In this sense, this approach to treating PTSD goes beyond psychotherapy or pharmacotherapy. Excessive focus on medical aspects, and ignoring other social (cultural, political, or economic) determinants of PTSD, can be misleading. For instance, in our study, we demonstrated that the development of PTSD in individuals who had lost a loved one as a result of war was different if such individuals were able to continue their education compared to those who did not have a chance to do so in the aftermath of war. In other words, being able to learn (whatever one’s education level) becomes one’s resource which can mitigate the negative effects of PTSD. A multi-dimensional approach to PTSD, taking into account both health and social aspects, will help develop social work practices not only through individual interventions but also by supporting individuals as members of their families, neighbourhoods, communities, and other social systems that might be important for them [71]. A study conducted in 2013–2014 among war veterans living in California showed that people suffering from increased PTSD were more likely than those with low PTSD to report problems with their partner/marital relationships and to recognise the negative impact of PTSD symptoms on the functioning of their children [72]. This suggests the necessity of taking into account the needs and problems of the social environment which is affected by the behaviour of persons suffering from PTSD. Our study shows that material security lowers PTSD, but only among people who have no children. On the one hand, having a family can provide protection against the negative effects of war stress. However, it can also create major mental strain and fear for the future of one’s children and one’s ability to provide them with safe living conditions, which in turn increases PTSD. The research shows that war veterans face a number of barriers to material security for themselves and their loved ones, including lack of stable employment, inability to manage their finances. Sometimes these problems are the source of homelessness [73]. The obtained results may perhaps be explained by the fact that persons experiencing PTSD, having a family and children to support, feel fear, anxiety about their future. Material security in their situation is not a factor reducing the severity of PTSD. The absence of children may be associated with less psychological strain. Of course, this cannot be concluded from the results obtained. These issues need to be further explored, though.

### 4.7. Study Limitations

There were several limitations of the study. Results may be skewed by disproportionate sample sizes in subgroups. The convenience sampling method used in the study results in an indeterminate probability of representativeness.

Due to the cross-sectional character of this study, our findings do not constitute a reliable basis for the identification of causal relationships between our variables. Another limitation to this study is PTSD measurement. Respondents can unconsciously associate trauma with war experiences, while the sources of PTSD can be connected with some other, or additional, experiences that have coincided with the war. For example, results of a study conducted in 2018 among civilians and military veterans of both formal and informal military organizations aged 60+, who had been involved in the American War in Vietnam (1965–1975), show that the loss of a loved one can contribute to the development of, or an increase in, PTSD. However, this correlation is weak, and the loss of family members needs to be analysed in isolation from other traumatic events occurring during war. In a way, the loss of loved ones is part of the war experience and nearly inseparably linked with it [74].

## 5. Conclusions

PTSD is the result of not only the violence and damage caused by war but also of other stressful circumstances associated with the social and financial conditions of life. Trauma-affected people need to be given opportunities to build up their strengths and increase their psychological resources towards well-being, e.g. in the area of education. Social security (e.g. health insurance or material security) should be taken into account when working with people affected by PTSD. Our findings can inform the goals behind therapeutic support for civilian participants of hostilities, and implications for social work. Social work professionals are encouraged to engage in direct questioning and to maintain a supportive and safe environment for participants in hostilities.

## Figures and Tables

**Table 1:** Parametric test results for PTSD score.

		*n*	*M*	*SD*	Value	*p*	Effect Size
Age	≤25	62	14.45	12.53	2.922	0.009 **	0.24
	26–30	63	20.87	14.63			
	31–35	62	22.52	12.35			
	36–40	43	22.19	11.88			
	41–45	29	24.52	16.81			
	46–50	27	21.63	13.05			
	>50	17	23.24	17.46			
Gender	Female	74	25.39	14.18	−3.420	0.001 **	0.45
	Male	232	19.13	13.57			
Civil status	Married/cohabitating	127	21.37	14.50	−0.459	0.647	-
	Single/separated/divorced/widow(er)	172	20.63	13.32			
Children	Yes	192	22.83	14.06	−3.719	0.000 ***	0.44
	No	116	16.88	12.83			
Education	Primary/secondary education	165	21.62	15.26	1.348	0.179	-
	Higher education	132	19.40	12.55			
Continuing education	Yes	68	14.90	13.58	3.983	0.000 ***	0.55
	No	240	22.33	13.58			
Place of residence	Village	67	25.94	13.68	3.555	0.000 ***	0.49
	City	236	19.23	13.63			
Material status	Bad	144	22.64	14.84	2.499	0.013 *	0.28
	Good	163	18.73	12.57			
Lost loved one	Yes	57	28.65	16.27	−5.090	0.000 ***	0.69
	No	245	18.61	12.67			
Distance from hostilities	≤500 km	71	21.11	13.49	0.340	0.712	-
	501–999 km	171	19.61	13.68			
	≥1000 km	41	20.78	15.38			
Health insurance	Yes	54	16.61	12.48	2.448	0.015 *	0.38
	No	255	21.65	13.98			
Savings	Yes	75	18.52	12.07	1.584	0.114	-
	No	233	21.42	14.33			
Material security	Yes	145	21.22	13.72	−0.688	0.492	-
	No	163	20.13	14.08			

* <0.05; ** <0.01; *** <0.001.

**Table 2:** Model summary for PTSD score: stepwise multiple regression analysis.

Model	*R*	*R* ^2^	Adjusted *R*^2^	*R*^2^ Change	*F* Change	*p*
1	0.286	0.082	0.078	0.082	24.106	0.000 ***
2	0.379	0.143	0.137	0.062	19.413	0.000 ***
3	0.429	0.184	0.175	0.041	13.412	0.000 ***
4	0.480	0.231	0.219	0.047	16.233	0.000 ***
5	0.508	0.258	0.244	0.027	9.720	0.002 **

** < 0.01; *** < 0.001.

**Table 3:** Multiple Regression Analysis Predicting PTSD score (final model).

	Unstandardized Coefficients		Beta	*t*	*p*	95% CI		Collinearity	
	B	S.E				Lower	Upper	Tol.	VIF
Constant	27.121	1.742		15.572	0.000 ***	23.692	30.550		
Lost loved one	7.543	1.951	0.208	3.867	0.000 ***	3.703	11.384	0.957	1.045
Place of residence	−1.972	0.385	−0.280	−5.126	0.000 ***	−2.729	−1.215	0.931	1.074
Gender	8.682	1.801	0.270	4.821	0.000 ***	5.136	12.228	0.889	1.124
Continuing education	−6.091	1.797	−0.185	−3.389	0.001 **	−9.630	−2.552	0.930	1.076
Health insurance	−6.333	2.031	−0.172	−3.118	0.002 **	−10.332	−2.334	0.911	1.097

** < 0.01; *** < 0.001.

## Data Availability

The datasets used and analysed during the current study are available from the corresponding author on reasonable request.
